# Systemic readiness and policy gaps for metabolic syndrome care in botswana prisons: A national WHO building blocks evaluation

**DOI:** 10.1016/j.hpopen.2026.100182

**Published:** 2026-09-08

**Authors:** Lebapotswe Bahumi Tlale, Lame Sharon Simon, Yogan Pillay, Debashis Basu

**Affiliations:** a Prison Health Services, Botswana Prison Service, Gaborone, Botswana; b DMed Public Health, University of Pretoria, Pretoria, South Africa; c Strategy and Transformation, Botswana Prison Service, Gaborone, Botswana; d Department of Public Health, University of Pretoria, Pretoria, South Africa; e Head of Department of Public Health medicine, University of Pretoria, Pretoria, South Africa

**Keywords:** Prison health, Metabolic syndrome, Noncommunicable diseases, Health systems, Health policy, Botswana

## Abstract

**Background:**

Metabolic syndrome is a growing global health challenge, affecting one in four adults worldwide, with prevalence in sub-Saharan Africa estimated at 20–30%. In Botswana, urban prevalence is 27%, reflecting a high burden of NCDs. Prisoners are particularly vulnerable due to overcrowding, poor nutrition, sedentary confinement, and limited access to health services.

**Objectives:**

To evaluate the systemic capacity, effectiveness, and readiness of Botswana's prison health care delivery mechanisms in managing metabolic syndrome, focusing on diagnostic, treatment, and continuity-of-care protocols.

**Methods:**

A descriptive cross-sectional study was conducted across all 20 prison health facilities in Botswana. A structured questionnaire adapted from the WHO Service Availability and Readiness Assessment (SARA) tool was administered to healthcare providers. Composite scores were generated to produce a Facility Readiness Index (FRI).

**Results:**

Service delivery and health information systems demonstrated moderate readiness, with most facilities maintaining chronic disease registers and reporting NCD statistics. Workforce shortages were pronounced, particularly in health posts, which relied heavily on single nurses. Medicines and equipment were moderately available, but frequent stock-outs of insulin, oral hypoglycemics, and inhalers undermined continuity of care. Governance and financing emerged as the weakest domains, with half of facilities lacking standard operating procedures and only 20% reporting dedicated budgets for NCD care. The overall FRI was 58%, indicating low-to-moderate systemic readiness.

**Conclusion:**

Botswana's prison health system demonstrates significant gaps in readiness for metabolic syndrome care. Strengthening diagnostic capacity, workforce training, medicine supply chains, and integration with national health systems is essential to safeguard prisoner health and broader public health outcomes.

## 1 Background

Metabolic syndrome (MetS) refers to a cluster of interrelated metabolic risk factors that increase the risk of cardiovascular disease and type 2 diabetes [1], [2], [3]. Diagnostic definitions vary, but most widely used criteria require the presence of at least three of the following five components: elevated waist circumference (abdominal obesity), elevated triglycerides, reduced high-density lipoprotein (HDL) cholesterol, elevated blood pressure, and elevated fasting plasma glucose [2].Globally, MetS affects approximately 25% of adults, with prevalence overall prevalence of MS in Africa was 32.4% [4], [5]. A 2021 study in Botswana found that metabolic syndrome affected 27% of adults, with semi-urban populations disproportionately impacted. In the same year, the prevalence of non-communicable diseases among adults was also estimated at around 27% [6], [7].

Globally, prison health has been recognized as a litmus test for a national health system resilience [8], [9]. The WHO and UNODC emphasize that prison health is public health, given the permeability between custodial settings and communities [8], [10], [11]. Incarcerated populations are not isolated; they cycle in and out of prisons, carrying with them communicable and non-communicable disease burdens that directly affect community health outcomes [12]. Prison populations are particularly vulnerable to MetS due to structural determinants such as overcrowding, poor nutrition, sedentary confinement, and limited access to health services [13]. Evidence from high-income countries shows that incarcerated individuals have disproportionately higher prevalence of MetS compared to the general population [14], [15]. For example, studies in the United States and United Kingdom found elevated rates of obesity, hypertension, and diabetes among prisoners, yet prison health systems remain under-resourced and poorly integrated with national health services [16], [17].

In Africa, most prison health research has historically focused on communicable diseases such as HIV, tuberculosis, and hepatitis [18]. However, emerging studies highlight the rising burden of NCDs in custodial settings [19]. A cross-sectional study in Mozambique reported hypertension prevalence of 36.9% and diabetes prevalence of 10.3% among inmates, with family history and female sex identified as significant risk factors [20]. Research from South Africa, Nigeria, and Kenya similarly indicates that prison health systems are ill-prepared to address NCDs, citing limited diagnostic capacity, frequent medicine stock-outs, and inadequate workforce training 21., 22., [21]. However, systematic evaluations of prison health system readiness for metabolic syndrome management are virtually absent in the African literature.

Despite these findings, no national-level evaluations of prison health system readiness for MS care have been published in the African context. This gap is particularly concerning given the permeability between prisons and communities through staff, visitors, and released inmates which makes prison health inseparable from public health. Evaluating systemic readiness for MS care in custodial settings therefore provides actionable evidence for governance, financing, and workforce reforms that can strengthen national NCD strategies and advance progress toward universal health coverage [12], [22], 25.. While the Botswana Prison Service (BPS) has a mandate to provide safe custodial care and rehabilitation, little is known about the systemic readiness of its health care delivery mechanisms to manage NCDs. Previous investigations into Botswana's health system have highlighted challenges in medicine supply chains, workforce shortages, and governance gaps [23]. Yet, no national level study has systematically assessed prison health care capacity for metabolic syndrome management. This study therefore, seeks to evaluate the systemic capacity, effectiveness, and readiness of Botswana's prison health care delivery mechanisms in managing metabolic syndrome, focusing on diagnostic, treatment, and continuity of care protocols. This study contributes to global debates on prison health as a litmus test for national health system resilience. By situating Botswana's experience within WHO's building blocks framework, the findings provide internationally comparable evidence for policy-makers in LMICs.

## 2 Materials and methods

### 2.1 Study design

A descriptive cross-sectional quantitative design was employed to systematically measure institutional capacity, service readiness, and functional performance across Botswana's prison health facilities. This design provides a snapshot of systemic readiness and enables comparative analysis across facilities without longitudinal follow-up.

### 2.2 Study Setting

The study was conducted across 20 prison health facilities serving 22 correctional institutions and a Centre for Illegal Immigrants in Botswana. Facilities range from clinics with admission capacity to health posts, each staffed by at least one registered nurse providing primary, clinical, and mental health care. Mochudi health post is the only facility without a registered nurse and manned by a safety, health and environment officer.

### 2.3 Study participants

The unit of analysis was the prison health service unit within each facility. A questionnaire using the WHO Health Systems Building Blocks was administered to health care workers in each facility. Inmate level data were not collected; the focus was on facility performance and system infrastructure.

### 2.4 Recruitment

Healthcare workers with experience in chronic disease management were identified through professional channels (staff meetings, email invitations, healthcare forums). Participation was voluntary, with informed consent obtained prior to questionnaire completion. Data collectors provided respondents with a brief, standardized explanation of metabolic syndrome and its component conditions prior to questionnaire administration, referencing the commonly used criteria (presence of three or more of abdominal obesity, elevated triglycerides, low HDL cholesterol, elevated blood pressure, and elevated fasting glucose). Respondents were instructed to answer facility-level questions with reference to routine practice and available resources rather than individual patient cases. This standard briefing aimed to reduce misclassification arising from variable clinical terminology among respondents.

### 2.5 Data collection

We adapted the World Health Organization's Service Availability and Readiness Assessment (SARA) tool to the prison health context to capture facility-level readiness across the six WHO health system building blocks (service delivery; health workforce; medicines and equipment; health information systems; leadership/governance; financing) [42]. The SARA tool provides standardized indicators for service availability and readiness and has been widely used to generate comparable facility-level metrics across settings [2, 42]. For this study we selected SARA items relevant to chronic disease management (screening, chronic disease registers, essential medicines and diagnostics, SOPs, and reporting) and supplemented them with prison-specific items (frequency of clinic days, task-shifting practices, and supply-chain experiences). The adapted questionnaire is provided in Supplementary Appendix A.

Although no formal pilot study was conducted prior to national deployment, the adapted instrument was reviewed by a panel of 5 prison officers (A public health Specialist, Nurse, Safety and Health officers and Statisticians) with experience in SARA-based assessments to ensure face validity and contextual relevance. The SARA tool and similar SARA-derived Facility Readiness Index approaches have been validated and used in multiple LMIC studies to assess readiness for NCD care, providing precedent for adaptation without a separate pilot when time or access constraints exist [43, 44]. Reliability was assessed through internal consistency checks (Cronbach's alpha) across domains. We acknowledge this as a limitation. A structured questionnaire, adapted from the World Health Organization's Service Availability and Readiness Assessment (SARA) tool, was deployed across all selected prison health facilities [42]. The instrument was designed to capture multiple dimensions of health system performance relevant to chronic disease care in custodial settings. It included items on facility identification and capacity, enabling documentation of the size, type, and operational characteristics of each health unit. Service availability and delivery were assessed to determine the range of chronic disease services offered, their accessibility to inmates, and the extent to which continuity-of-care protocols were in place.

The questionnaire also examined workforce and training, focusing on the number, qualifications, and preparedness of healthcare staff to manage metabolic syndrome and related conditions. Essential medicines and equipment were evaluated to establish the availability of diagnostics, treatment supplies, and technologies required for effective chronic disease management. In addition, the tool assessed health information systems, including the presence of documentation practices, reporting mechanisms, and integration with national health databases. Finally, domains on leadership, governance, and financing were included to explore oversight structures, resource allocation, and systemic factors influencing the delivery of prison health services.

### 2.6 Data analysis

Data were analysed using descriptive statistical techniques, including the calculation of means, and proportions, to provide a clear overview of facility performance. Comparative methods were employed to examine differences across facility types and regions, allowing for the identification of systemic patterns and disparities. Each indicator within the assessment tool was rated on a standardized scale ranging from 0 to 3, where a score of 0 indicated that the service or resource was not available and a score of 3 reflected full functionality.

Each indicator was scored on a standardized ordinal scale and normalized to a 0–100 scale to produce domain-specific readiness scores. Domain scores were averaged to compute an overall Facility Readiness Index (FRI), consistent with prior SARA-based composite approaches used to benchmark facility readiness for chronic disease services. Facilities were categorized as high (≥80%), moderate (60–79%), or low (<60%) readiness to facilitate interpretation and policy prioritization. The FRI approach allows comparison across domains and with other SARA-based studies. This classification provided a structured framework for benchmarking performance and identifying priority areas for intervention.

## 3 Results

The results of this national cross-sectional evaluation are presented according to the six WHO health system building blocks, providing a structured overview of systemic readiness for metabolic syndrome care within Botswana's prison health system. Data were collected from all 20 prison health facilities serving 22 correctional institutions and a Centre for Illegal Immigrants, enabling comprehensive coverage of service delivery, workforce capacity, medicines and equipment availability, health information systems, and governance and financing mechanisms. The findings highlight both strengths and weaknesses across domains, offering insight into the extent to which prison health services are prepared to manage non-communicable diseases in custodial settings. Tables and narrative summaries are used to illustrate facility-level performance, comparative differences between clinics and health posts, and systemic gaps that undermine continuity of care. This structured presentation provides a clear benchmark for assessing readiness and identifying priority areas for reform.

### 3.1 Service availability and delivery

Table 1 presents the availability of key service delivery indicators across clinics and health posts within Botswana's prison health system. The indicators assessed include the presence of chronic illness clinics, routine screening for metabolic syndrome, and referral protocols for metabolic syndrome cases. The comparison highlights differences in service provision between the two facility types, with clinics generally reporting higher availability of referral protocols, while health posts demonstrated stronger performance in routine screening. Statistical testing using Pearson's chi-square showed no significant differences between facility types across all three indicators, suggesting that service availability challenges are systemic rather than facility-specific. Effect sizes were calculated to assess the magnitude of differences; all values indicated small effects, consistent with non-significant chi-square results.

**Table 1:** Service Availability Indicators by Facility Type.

Indicator	Clinic		Health Post		Total (All Facilities)		χ^2^ (*p*-value)
	Yes (%)	No %	Yes (%)	No (%)	Yes (%)	No (%)	
Chronic illness clinic available	6 (75%)	2 (25%)	6 (50%)	6 (50%)	12 (60%)	8 (40%)	1.250 (0.264)
Routine screening conducted	5 (62.5%)	3 (37.5%)	10 (83.3%)	2 (16.7%)	15 (75%)	5 (25%)	1.999 (0.292)
Referral protocols present	7 (87.5%)	1 (12.5%)	9 (75%)	3 (25%)	16 (80%)	4 (20%)	0.469 (0.494)

### 3.2 Frequency of metabolic syndrome services

Metabolic syndrome services were provided per week across clinics and health posts. The distribution shows considerable variation in service availability (Table 2). Clinics reported provision ranging from no services (28.5%) to daily services (28.5%), with smaller proportions offering services once or five times per week. Health posts demonstrated stronger daily coverage, with half (50%) reporting seven-day service provision, though some facilities still offered limited or no services. Statistical testing using Pearson's chi-square indicated no significant difference between clinics and health posts in terms of service frequency (χ^2^ = 2.083, *p* = 0.720), suggesting that variability in service provision is widespread across facility types rather than confined to one category.

**Table 2:** Frequency of Metabolic Syndrome Services per Week.

Facility	0 days	1 day	5 days	7 days	Total
Clinic	2 (28.5%)	1 (14.3%)	2 (28.5%)	2 (28.5%)	7
Health Post	1 (12.5%)	1 (12.5%)	2 (25%)	4 (50%)	8
Total (All Facilities)	3 (15%)	2 (10%)	4 (20%)	6 (30%)	15

*Pearson χ*^*2*^ *= 2.083, p = 0.720.*

### 3.3 Health workforce and training

The composition of health personnel and the presence of task-shifting practices across clinics and health posts (Table 3). The data show that clinics were more likely to have diverse staffing arrangements, including doctors, nurses, and allied health professionals, whereas health posts were predominantly staffed by single nurses. Task shifting was reported in 62.5% of clinics compared to 33.3% of health posts, reflecting efforts to mitigate workforce shortages. Staffing gaps were widely acknowledged, with 88.9% of facilities reporting insufficient personnel as a barrier to effective metabolic syndrome service delivery. Although clinics demonstrated relatively greater workforce diversity and adoption of task-shifting, the chi-square test indicated no statistically significant difference between facility types (χ^2^ = 1.650, *p* = 0.199). This suggests that workforce challenges are systemic across the prison health system, with both clinics and health posts constrained by limited human resources.

**Table 3:** Workforce Composition and Task Shifting.

Indicator	Clinic	Health Post	χ^2^ (p-value)
Diverse staffing (doctor + nurses + allied staff)	Present in 6 facilities	Rare (mostly single nurse)	–
Task shifting in place	5 (62.5%)	4 (33.3%)	1.650 (0.199)
Reported staffing gaps	8 (88.9%)	1 (11.1%)	–

### 3.4 Essential medicines and equipment

The availability of essential medicines and diagnostic equipment varied across prison health facilities, with notable supply chain instability undermining continuity of care. Antihypertensives were widely available in both clinics (100%) and health posts (66.7%), yet half of health posts reported more than three stock-outs in the past year (Table 4). Insulin and oral hypoglycaemics were particularly inconsistent, available in only 41.7% of health posts and 87.5% of clinics, with recurrent shortages reported in nearly half of facilities. Inhalers were present in most sites, but frequent interruptions in supply were observed, especially in clinics where half experienced more than three shortages. Diagnostic equipment such as blood pressure monitors was consistently available, though glucometer functionality was compromised by insufficient test strips in over 40% of health posts and 62.5% of clinics. These findings underscore that while basic infrastructure exists, recurrent stock-outs of critical medicines and consumables pose a major barrier to effective metabolic syndrome management in custodial settings.

**Table 4:** Availability and Stock-Outs of Essential Medicines and Equipment.

Health facility type	Item	Availability		Total	Stock-outs			Total
Health Post		Yes (%)	No (%)		Never	Once	>3 times	
	Antihypertensives	8 (66.7)	4 (33.3)	12	3 (25.0)	3 (25.0)	6 (50.0)	12
	Insulin/oral hypoglycemics	5 (41.7)	7 (58.3)	12	4 (33.3)	4 (33.3)	5 (41.7)	12
	Inhalers	10 (83.3)	2 (16.7)	12	4 (33.3)	5 (41.7)	3 (25.0)	12
	BP monitors	12 (100.0)	–	12	2 (16.7)	10 (83.3)	–	12
	Glucometers	9 (75.0)	3 (25.0)	12	Functional: 7(58.3)	Insufficient strips: 5 (41.7)	–	12
Clinic	Antihypertensives	8 (100.0)	–	8	2 (25.0)	4 (50.0)	2(25.0)	8
	Insulin/oral hypoglycemics	7 (87.5)	1 (12.5)	8	3 (37.5)	3 (37.5)	2 (25.0)	8
	Inhalers	6 (75.0)	2 (25.0)	8	–	4 (50.0)	4 (50.0)	8
	BP monitors	7 (87.5)	1 (12.5)	8	–	7 (87.5)	1 (12.5)	8
	Glucometers	7 (87.5)	1 (12.5)	8	Functional: 3(37.5)	Insufficient strips: 5 (62.5)	–	8

### 3.5 Health information systems

The majority of facilities reported maintaining chronic disease registers, with all health posts (100%) and most clinics (87.5%) documenting patient data (Table 5). Availability of NCD statistics for the last quarter was similarly high, reported by 87.5% of clinics and 83.3% of health posts. However, the adoption of digital health records remained limited, with only 29.1% of clinics and 25% of health posts reporting use of electronic systems. Chi-square testing showed no statistically significant differences between clinics and health posts across all indicators, suggesting that strengths in record-keeping and reporting, as well as weaknesses in digitalization, are consistent across facility types. These findings highlight that while basic health information systems are in place, reliance on paper-based documentation constrains integration with national databases and limits opportunities for real-time monitoring and continuity of care.

**Table 5:** Health Information System Indicators.

Indicator	Clinic		Health Post		Total (All Facilities)		χ^2^ (p-value)
	Yes (%)	No (%)	Yes (%)	No (%)	Yes (%)	No (%)	
Chronic disease register maintained	7 (87.5%)	1 (12.5%)	12 (100%)	0	19 (95%)	1 (5%)	1.579 (0.209)
NCD statistics available (last quarter)	7 (87.5%)	1 (12.5%)	10 (83.3%)	2 (16.7%)	17 (85%)	3 (15%)	0.065 (0.798)
Digital health records in use	3 (29.1%)	5 (70.8%)	3 (25%)	9 (75%)	6 (30%)	14 (70%)	0.357 (0.550)

### 3.6 Leadership, governance, and financing

The presence of standard operating procedures (SOPs) and protocols was reported in half of both clinics and health posts, indicating limited institutional standardization (Table 6). Similarly, inclusion of NCD indicators in performance reviews was reported by 75% of facilities across both types, suggesting some alignment with monitoring frameworks but not universal coverage.

**Table 6:** Governance and Financing Indicators by Facility Type.

Indicator	Clinic		Health Post		Total (All Facilities)		χ^2^ (p-value)
	Yes (%)	No (%)	Yes (%)	No (%)	Yes (%)	No (%)	
SOPs/protocols present	4 (50%)	4 (50%)	6 (50%)	6 (50%)	10 (50%)	10(50%)	0.000 (1.00)
NCD indicators in performance review	6 (75%)	2 (25%)	9 (75%)	3 (25%)	15 (75%)	5 (25%)	0.000 (1.00)
Dedicated NCD budget	0	8 (100%)	4 (33.3%)	8 (66.7%)	4 (20%)	16 (80%)	3.333 (0.68)
Financing/supply chain challenges	7 (87.5%)	1 (12.5%)	6 (54.5%)	5 (45.5%)	13 (60%)	6 (35%)	2.328 (0.127)

Dedicated budgets for NCD care were notably absent in clinics (0%), while only one-third of health posts (33.3%) reported having such allocations. This highlights a critical gap in financial planning and prioritization of chronic disease management. Financing and supply chain challenges were widely reported, affecting 87.5% of clinics and 54.5% of health posts. Although the chi-square tests revealed no statistically significant differences between facility types, the findings underscore systemic weaknesses in governance and financing that cut across the prison health system.

These results suggest that while performance monitoring is partially integrated, the absence of dedicated budgets and persistent supply chain challenges undermine the sustainability of metabolic syndrome care. Strengthening governance frameworks and establishing reliable financing mechanisms are essential to ensure continuity and equity in service delivery.

### 3.7 Composite facility readiness index (FRI)

Table 7 summarizes the composite Facility Readiness Index (FRI) across the six WHO health system building blocks. The analysis shows that service delivery (68%), medicines and equipment (62%), and health information systems (72%) achieved moderate readiness levels, reflecting partial capacity to support metabolic syndrome care. In contrast, workforce (55%), governance (50%), and financing (40%) scored low, highlighting systemic weaknesses in human resources, institutional oversight, and financial sustainability.

**Table 7:** Composite Facility Readiness Index (FRI) by WHO Building Block.

Building Block	Mean Score (%)	Readiness Level
Service Delivery	68	Moderate
Workforce	55	Low
Medicines & Equipment	62	Moderate
Health Information Systems	72	Moderate
Governance	50	Low
Financing	40	Low
Overall FRI	58	Low–Moderate

The overall FRI score of 58% indicates low-to-moderate readiness of Botswana's prison health system for metabolic syndrome management. This composite measure underscores that while certain domains particularly health information systems are relatively stronger, critical deficits in workforce, governance, and financing undermine the system's ability to deliver consistent and equitable care.

## 4 Discussions

This study provides the first national-level evaluation of systemic readiness for metabolic syndrome (MetS) care in Botswana's prison health system. MetS is defined as the clustering of at least three of five cardiometabolic risk factors abdominal obesity, hypertension, elevated fasting glucose, high triglycerides, and low HDL cholesterol that collectively increase risk for cardiovascular disease and type 2 diabetes [2], [3]. By situating findings within this definition, our results highlight how systemic weaknesses directly undermine the prevention, diagnosis, and management of these precursor conditions.

Overall systemic capacity remains low to moderate, with critical weaknesses in workforce, governance, and financing. While service delivery and health information systems demonstrated moderate readiness, variability in service frequency and routine screening limited continuity of care. Workforce shortages were pronounced, particularly in health posts reliant on single nurses, and training in MetS management was limited. Medicines and diagnostic equipment were moderately available, yet frequent stock-outs of insulin, oral hypoglycemics, and inhalers disrupted continuity of care. Health information systems were relatively stronger, with most facilities maintaining chronic disease registers and reporting NCD statistics, though reliance on paper-based systems constrained integration with national databases. When we refer to “readiness” in this study, we mean facility-level capacity to provide the structural and process elements necessary for prevention, diagnosis, treatment, and continuity of care for metabolic syndrome and its component conditions (hypertension, dyslipidaemia, diabetes, and obesity-related care). The FRI captures availability of services, trained personnel, essential medicines and diagnostics, information systems, and governance/financing arrangements that together enable effective chronic disease management. Weaknesses in workforce, governance, and financing therefore indicate systemic barriers to consistent screening, diagnosis, and long-term management of metabolic syndrome and its components rather than a direct measure of disease prevalence. Strengthening these domains (for example, ensuring reliable supply chains for antihypertensives and insulin, expanding workforce training, and integrating prison health reporting with national NCD surveillance) would improve the system's ability to prevent and manage metabolic syndrome and reduce downstream cardiovascular and diabetes complications.

Governance and financing emerged as systemic bottlenecks. Half of facilities lacked standard operating procedures, only 20% reported dedicated budgets for NCD care, and widespread supply chain challenges were reported. These deficits constrain the effectiveness of otherwise moderately functional domains. Without leadership commitment and ring-fenced financing, continuity of MetS care cannot be sustained, and equity in service delivery remains compromised. The Botswana findings align with evidence from other African contexts. In Nigeria, weak governance and poor integration of prison health into national systems have left NCD management fragmented [21]. In Kenya, evaluations highlight inadequate workforce training and the absence of standardized protocols for chronic disease care. In Mozambique, cross-sectional data revealed high prevalence of hypertension (36.9%) and diabetes (10.3%) among inmates, underscoring the burden of cardiometabolic risk factors in custodial settings [20]. Collectively, these studies reinforce that prison health systems across Africa remain poorly prepared for the growing burden of MetS and NCDs, despite WHO and UNODC recognition that prison health is public health [8], [10].

Policy implications from this study are clear. Integrating prison health into Botswana's national NCD strategy is essential to ensure equity and sustainability. Strengthening supply chains for essential medicines, particularly insulin and antihypertensives, will improve continuity of care. Workforce training and task-shifting must be expanded to address chronic disease management, while dedicated financing streams and governance mechanisms are needed to support service delivery. Enhancing digital health information systems would also improve documentation, monitoring, and integration with national databases, thereby strengthening continuity of care. These reforms would not only improve prisoner health outcomes but also contribute to national health system resilience and public health equity [24].

Our findings point to five priority policy actions. First, integrate prison health explicitly into Botswana's national NCD strategy with a ring-fenced budget line for custodial NCD care. Second, establish a formal supply-chain agreement between the Botswana Ministry of Health and the Botswana Prison Service to eliminate recurrent stock-outs of insulin and essential medicines. Third, scale task-shifting and targeted training for nurses and health officers to expand capacity for chronic disease management. Fourth, pilot digital chronic disease registers in selected facilities to enable continuity of care and integration with national health information systems. Fifth, formalize referral pathways and discharge planning to ensure continuity of care on release, thereby protecting both prisoner and community health. The findings of this study have important implications beyond Botswana, reflecting systemic challenges in prison health systems worldwide. Non-communicable diseases (NCDs), including metabolic syndrome, are rising rapidly across low- and middle-income countries, yet custodial settings remain under-prioritized in health system planning.

This study provides the first national-level evaluation of systemic readiness for metabolic syndrome care in Botswana's prison health system. While service delivery and health information systems demonstrated moderate readiness, overall systemic capacity remains low to moderate, with critical weaknesses in workforce, governance, and financing. These findings resonate with international evidence: weak governance in Nigeria, inadequate workforce training in Kenya, and high cardiometabolic burden in Mozambique all point to systemic neglect of NCDs in custodial settings 25., [23], [24], [25].

Beyond Botswana, the implications are global. Prisons are permeable institutions where health challenges extend into communities through staff, visitors, and released inmates. Weak readiness for NCD management therefore undermines both national resilience and global public health equity. The WHO Prison Health Framework emphasizes integration with national health systems and accountability mechanisms, while SDG 3.8 calls for universal health coverage, including equitable access to essential medicines and services for custodial populations [8], [25], 29.. By applying the WHO health system building blocks and developing a Facility Readiness Index (FRI), this study offers a replicable benchmark for evaluating custodial health systems globally [8], 29.. In this way, Botswana's experience contributes to the global literature on health equity, systems resilience, and preparedness for NCDs in marginalized populations. Strengthening prison health systems is not only a matter of human rights but also a strategic investment in national and international public health security.

The study has several strengths and limitations. It provides comprehensive national coverage by including all 19 prison health facilities, offering a holistic picture of systemic readiness. The use of the WHO health system building blocks framework ensures comparability with international assessments, while the development of the Facility Readiness Index (FRI) provides a standardized benchmark for monitoring progress and guiding policy interventions. However, this study has several limitations. First, the questionnaire was adapted from the WHO SARA tool for the prison context but was not piloted in a separate pilot study prior to national deployment. While the adaptation was reviewed by subject-matter experts and SARA-based composite indices have precedent in LMIC research, the absence of a formal pilot may have limited our ability to detect ambiguous items or to quantify measurement reliability in this specific custodial setting. Second, the assessment relied on facility-level self-report by healthcare staff rather than direct observation or patient-level clinical measurements; this may introduce reporting bias, particularly if respondents interpreted questions in the context of general chronic disease care rather than metabolic syndrome specifically. Third, diagnostic thresholds and clinical understanding of metabolic syndrome may vary among respondents; although we provided a brief definition of metabolic syndrome to respondents during data collection, differential interpretation remains possible. Despite standardized briefing, residual bias may persist if respondents conflated metabolic syndrome with general NCD management. This self-report nature introduces potential misclassification. These limitations should be considered when interpreting the FRI and domain scores. Future work should include piloting the adapted instrument, triangulating facility reports with direct observation and patient-level data, and validating the FRI against clinical outcomes limitations must be acknowledged. The cross-sectional design provides only a snapshot in time and does not capture longitudinal changes. Reliance on self-reported data from facility staff introduces the possibility of reporting bias. Missing data, particularly on referral frequency and supply chain indicators, limited the analysis of continuity-of-care. Finally, the study focused on facility readiness rather than direct health outcomes among prisoners, which may have provided additional insights into the effectiveness of service delivery.

## 5 Conclusion

This study provides the first national-level evaluation of systemic readiness for metabolic syndrome care in Botswana's prison health system. Overall capacity was low to moderate, with critical weaknesses in workforce, governance, and financing. Addressing these gaps is urgent not only for custodial equity but also for public health, given the permeability between prisons and communities. The Facility Readiness Index offers a replicable benchmark for other low- and middle-income countries, aligning with WHO and UNODC recognition that prison health is public health. Policy-makers should prioritize integration of prison health into national NCD strategies, dedicated financing and governance mechanisms, and investment in workforce development and supply chain stability to advance universal health coverage and safeguard vulnerable populations.

## CRediT authorship contribution statement

**Lebapotswe Bahumi Tlale:** Writing – review & editing, Writing – original draft, Visualization, Validation, Supervision, Software, Resources, Project administration, Methodology, Investigation, Formal analysis, Data curation, Conceptualization. **Lame Sharon Simon:** Writing – review & editing, Visualization, Validation, Software, Resources, Methodology, Formal analysis, Data curation, Conceptualization. **Yogan Pillay:** Writing – review & editing, Visualization, Validation, Supervision, Resources, Project administration, Methodology, Investigation, Conceptualization. **Debashis Basu:** Writing – review & editing, Visualization, Validation, Supervision, Software, Resources, Project administration, Methodology, Investigation, Formal analysis, Data curation, Conceptualization.

## Consent for publication

Not applicable.

## Ethics approval and consent to participate

This study was approved by the University of Pretoria Faculty of Health Sciences Research Ethics Committee (Reference number: 25/2025) and by the Botswana Ministry of Health Institutional Review Board.

## Funding

This study did not receive external funding. It was supported by institutional resources from the Botswana Prison Service and academic facilitation from the 10.13039/501100001343University of Pretoria and University of Botswana. Institutional support did not influence analysis or reporting.

## Declaration of competing interest

The authors declare that they have no known competing financial interests or personal relationships that could have appeared to influence the work reported in this paper.
